# Cultural Competence Guides for COVID-19 Messaging in Hispanic Communities

**DOI:** 10.7759/cureus.40820

**Published:** 2023-06-22

**Authors:** Madeline Huff, Shuchita V Jhaveri, Ayesha Khan, Lina Pedraza, Maria Pesantez Borja, Daniela Santos Cantu, Chelsea Chang

**Affiliations:** 1 Internal Medicine, University of Texas Rio Grande Valley School of Medicine, Edinburg, USA; 2 Family Medicine, University of Texas Rio Grande Valley School of Medicine, Edinburg, USA

**Keywords:** interdisciplinary, rio grande valley, hispanic, public health messaging, covid-19, cultural competence

## Abstract

Purpose: The Rio Grande Valley in South Texas comprises 5% percent of Texas’s population yet 17%of Texas’s COVID-19 deaths. We aimed to address underlying mistrust and systemic racism in our Hispanic community that contributes to health inequities by developing a cultural competence guide for public health messaging.

Methods: We employed a mixed method design (e.g., focus groups, surveys, interviews) to develop and implement a cultural competence guide in an iterative community-informed process. We created a general cultural competence guide, one for the Hispanic community and one for the hard-of-hearing community.

Results: Our cultural competence guides provide an interpretation as to whether the message is culturally competent or requires revisions. The guides have the following five categories: content and clarity, emotions and values, audience and inclusivity, call to action, and gestalt.

Conclusions: The Hispanic community needs more culturally competent public health messaging to address a key root cause of health inequities surrounding COVID-19. Our novel, concise guides can help organizations and individuals seeking to create culturally sensitive and, therefore, more effective public health messaging for Hispanic or deaf and hard-of-hearing communities.

## Introduction

The COVID-19 pandemic has exposed dire health inequities, systemic racism, and underlying mistrust. Systemic racism perpetuates an unequal distribution of opportunities, goods, and services based on race [1]. These inequalities extend into healthcare with health inequalities disproportionately affecting minorities such as Black Americans and Hispanics [2-3]. Comparing the burden of disease for COVID-19 between racial groups, Black Americans had a mortality rate 2.1 times higher than Whites, and Latinos experienced 4.6 times as many hospitalizations as Whites [4]. These findings can be attributed to social and economic inequalities that exist in society and in our healthcare system [5].

We at the University of Texas Rio Grande Valley School of Medicine (UTRGV SOM) serve a four-county area of 1.4 million residents along the southern US-Mexico border, the Rio Grande Valley (RGV). The RGV comprises 5% of Texas’s population, has a population that is 95% Hispanic, and had a staggering 17% of COVID-19 deaths across the state [6]. The RGV has the highest rates of obesity and diabetes nationwide which have compounded the impact of COVID-19 [7-8]. Another challenge is that 40% of our population are predominately or solely Spanish speakers. These COVID-19 inequities are rooted, among other factors, in systemic racism. Moreover, the population’s long-standing mistrust of health systems, including concerns surrounding immigration policy, deters many from seeking care even when urgently needed.

A lack of culturally sensitive information in healthcare further contributes to these inequalities [9-10]. Culturally sensitive care comes with cultural competence and cultural humility in the workplace. Cultural competency is a set of behaviors, attitudes, and policies that allow professionals to work in cross-cultural settings [11]. It is particularly useful in treating different minority populations as it complements patient-centered care. In healthcare, cultural competency in conjunction with patient-centered care helps providers identify the various cultural barriers to care in addition to identifying opportunities to improve care [12]. Cultural competency at the level of the healthcare provider and health systems has a positive impact on the quality of care. Studies show that as healthcare provider cultural competency increased, patient satisfaction among minority groups also increased [13]. As healthcare providers become more mindful of the cultural influences on health, healthcare disparities begin to decline. Healthcare providers were able to empower their patients, and as a result, patients participated in more screening tests to prevent disease [14]. The culturally competent approach to healthcare intervention is particularly important during the COVID-19 pandemic as it helps build a relationship between healthcare providers and minority populations.

We propose addressing underlying mistrust and systemic racism through an interdisciplinary learner-led, community-engaged, educational public health campaign targeting Spanish speakers and the 11,000 deaf and hard of hearing (HOH) individuals in the RGV. We aimed to develop and implement cultural competence guides for public health messages for Hispanic and HOH populations. To our knowledge, this is the first national attempt to develop a guide and rubric on culturally competent messaging for the Hispanic and HOH and deaf communities.​

## Materials and methods

Faculty and students from the UTRGV SOM, Graduate Medical Education (GME), and School of Social Work (SSW) gathered with safety-net clinic partners and developed goals and objectives for the project with the support of the AAMC Nurturing Experiences for Tomorrow's Community Leaders (NEXT) Award. Our interdisciplinary team engaged with community partners employing a mixed method design including focus groups, surveys, and qualitative interviews to explore barriers to culturally competent public health messaging. Our team developed and implemented a general cultural competence guide and then further developed one for the Hispanic community and one for the HOH community in an iterative community-informed process. Our project received UTRGV Institutional Review Board approval.

Exploring barriers to culturally competent public health messaging through community engagement

Surveys

We distributed surveys to the clinic leaders at our community partners at El Milagro Clinic and Hope Family Health Center to explore barriers to culturally competent public health messaging. The survey took approximately 15 minutes to complete with a total of 14 questions. We provided a consent form including that participation in this survey was voluntary and involved minimal risk, and participants had to be at least 18 years or older to participate. The survey inquired about the demographics of the clinic patients, insurance status, number of household members, and the COVID-19 risk of the clinic’s patients. We asked clinic leaders about the cultural values/themes that were present in their patients, where patients received most of their healthcare information and the relative hesitancy of patients regarding the annual influenza vaccine and COVID-19 vaccine.

We distributed the same 14-question survey to leaders of the HOH community. We also asked HOH leaders about the community’s communication preference (ASL, lip reading, amplification) and how many people in the community can understand ASL.

Qualitative Interviews

Via videoconferencing due to the pandemic restrictions, the interdisciplinary team held qualitative interviews with community safety-net clinic leaders and HOH leaders. We explored the results of their surveys and examples of successes and failures of public health messaging in COVID-19 in our communities. We explored the barriers and facets of culturally competent public health messaging in COVID-19 in the Hispanic community and the HOH community. We had open-ended discussions after watching and reading various messaging examples intended to be designed for our communities.

Focus Groups

Focus groups were held with patients of our safety-net clinics. We employed social distancing and masking in person. Our focus group contained ten adult participants, male and female, and was conducted in Spanish by native Spanish speakers of our team to match the language preference of our target audience.

We had a drafted general cultural competency guide which we used to explore the patients’ perceptions of public health messages. We showed preselected public health messages and allowed for both open-ended and specific responses from the audience. We asked what they liked about the message, what they didn’t like, and how they would summarize the message in their own words, among other questions.

Developing and implementing cultural competency guides and rubrics

General Guide

The interdisciplinary team performed a literature review on culturally sensitive, culturally competent public health messaging. The surveys and qualitative interviews were then used to develop the first draft of our one-page cultural competency guide and rubric. We aimed to have categories and descriptions within each category of what to do and what not to do with a final assessment to assess cultural competency. We planned for an iterative process after reviewing the guide with various stakeholders including applying to patients, public health experts, HOH members, clinic leaders, and our interdisciplinary team.

Hispanic Guide

After the development of the general guidelines described above, we narrowed our perspective to Hispanic communities and strived to find common cultural values and keys to culturally competent public health messaging for this diverse community. We employed the focus groups above and had an iterative process to develop this guide. We implemented the guide with our team members and other experts and explored areas of ambiguity or limitations.

HOH Guide

After the development of the general guidelines described above, our team did a literature review to better understand the barriers facing the HOH community in public health messaging. We had qualitative interviews and surveys with HOH leaders in our community and developed our HOH guide with their input, review, and guidance.

## Results

Exploring barriers to culturally competent public health messaging through community engagement

Surveys

**Figure 1:**
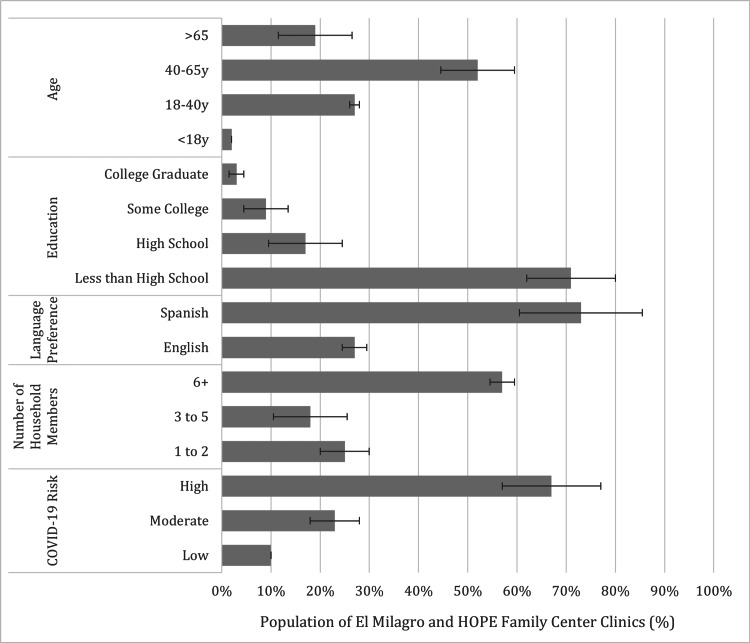
Safety net clinics survey respondents at El Milagro clinic and HOPE Family Center clinic over average patient age, education level, language preference, number of household members, and COVID-19 risk

Clinic community leaders completed the survey describing the patient population they treat. The patient population was 97% Hispanic/Latino with a 73% language preference for Spanish and a 98% uninsured status (Figure 1). Fifty-three percent About 53% were 40-65 years old, 28% were above 65 years old, 19% were 18-40 years old, and 2% were under 18 years old. Fifty-five percent identified as female, 40% identified as male, and 5% identified as transgender/non-binary/other. Seventy-one percent had no formal education or education less than the 12th grade, 17% had a high school diploma or GED, 9% had some college background, and 3% were college graduates. Fifty-seven percent live in a household with six people, 18% live in a household size of three to five people, and 25% live in a household with one to two people.

Sixty-seven percent were at high risk for COVID-19 with multiple underlying risk factors, including but not limited to obesity, hypertension, and diabetes. Ten percent were low-risk with no underlying risk factors, and 23% had only one underlying risk factor. Most of their healthcare information came from social media (Facebook, Instagram, etc.) as well as family members and friends. Twenty-five percent expressed hesitancy toward receiving their annual flu vaccination, while 35% expressed hesitancy toward receiving the COVID-19 vaccination.

The top cultural values for this patient population were identified as “familismo” (family involved in medical decisions), “personalismo” (personability or formal friendliness of healthcare provider), “jerarquismo” (respect for authority figures), and “espiritismo” (belief that good and evil spirits can affect health). Regarding what aspects of public service announcements (PSAs) need improvement to be more effective with their patients, clinic leaders identified that PSAs need clarity of message and availability in the patient’s preferred language.

In surveying the local HOH and deaf community experts, we had two experts participate who were also themselves members of the HOH community. The results of the survey showed that 50% of the local HOH community understands ASL, 70% use a combination of ASL and lip-reading, 30% use lip-reading alone, and 68% rely on amplification. The cultural values that were deemed as most important were collectivism and communication inclusivity.

Qualitative Interviews

HOH leaders voiced that public health messages targeting the HOH and deaf community should use a combination of ASL, open captions, and voice-over while always remembering linguistic fluency is critical. Credible sources should be used in terms of both organization skills and credibility of the person delivering the message who ideally would be a certified interpreter and someone who is part of the community.

Focus Groups

The outcome of the focus groups in evaluating public health messages delivered via social media, radio, and video showed that patients at the HOPE and El Milagro clinics responded most favorably to happy and concise public health messaging as opposed to more fearful or sad messaging tactics. Content and clarity were important in creating a strong message that presented accurate facts based on credible sources that were displayed in an organized and pleasing array. Additionally, emotions and values were a critical component that aimed to generate a strong narrative incorporating commonly shared beliefs and attitudes to induce a strong feeling of inspiration, hope, fear, and/or sadness. This emotional impact is intended to incorporate the cultural values of the target audience in a believable and engaging way. PSAs were also assessed for levels of demographic and psychographic inclusivity, such as age, sex, race, ethnicity, socioeconomic status, educational level, values, and lifestyle. This also includes whether the PSAs were in the target audience's primary language. Ultimately, the public health messages needed to invoke change and show that viewers can make a difference with their actions.

Developing and implementing cultural competency guides and rubrics

General Guide

Compiling lessons learned from the didactics, surveys, qualitative interviews, and patient-centered focus groups, we developed the general cultural competency guide (Appendix 1). The process for each guide involved at least five formal iterations: pre-focus group, post-focus group, post-community leader review, post-public health expert review, and post-implementation review. We will refer to the guide and rubric solely as a guide from here forward for simplicity.

Each guide assessed five major components for an effective culturally competent message: content and clarity, emotions and values, audience and inclusivity, call to action, and gestalt. We developed descriptions in each component for items in the “do” column and the “don’t do” column for a user-friendly and concise guide. To produce a quantitative outcome, we assigned a points system that is explained in all three guides, with each score being out of 25 points. The points interpretation is as follows: 0-14 points means major recommendations to the PSA are recommended, 15-19 points means minor revisions are recommended, and 20-25 points means the PSA was a culturally competent message.

We noted messages ideally should always be tested with the priority population through qualitative methods with focus groups or listening sessions. We found that many small organizations and individuals did not have the resources to host focus groups for their public health messaging. Therefore, we also allow and encourage the guide to be used without the requirement of a focus group to increase access to culturally competent messaging. All three guides are in a ready-to-print one-page format in Appendices 1-3.

Hispanic Guide

For the cultural competency guide for Hispanic communities, we started with the general cultural competency guide and began to identify features more specific to the Hispanic population, which is albeit still very diverse (Table 1, Appendix 2). We found many aspects to be unchanged from the general cultural competency guide, including a call to action and gestalt components. The aspects unique to the Hispanic community guide are detailed below.

**Table 1:** Rubric and guide on cultural competence in public health messaging (Hispanic community)

Main rubric Components:	Do (1 point per box except the last two are 5 points each)	Don't
Content and clarity	Strong and consistent message. Accurate grammar including proper use of accents. Accurate facts presented by credible/trustworthy sources like religious leaders, community leaders, celebrities, or sports athletes. Organized, tidy, pleasing, and symbolic colors (like colors of the flag or bright colors) are used. High-quality attractive graphics	Unclear and weak message. Spelling and grammatical errors. Questionable information or lack of trustable sources. Cluttered visuals or poor color scheme. Low-quality displeasing graphics
Emotions and values	Strong narrative that emphasizes emotion, Draws upon commonly shared beliefs and attitudes held among the audience including Espiritismo (the belief that evil/good spirits affect health) or superstition. Uses storytelling and emotional devices in a believable and engaging way Induces strong feelings of inspiration, hope, fear, and/or sadness. Hones in on the cultural values of the target audience especially the importance of religion, friendship, Familismo, and Jerarquismo (family and hierarchy)	Focuses on cold data or abstract concepts. Detached from an individual story or detached from the target audience. Does not employ emotional devices or is based on scare tactics alone. Does not induce feelings or is not engaging. Does not convey attitudes, values, and beliefs shared by the audience
Audience and inclusivity	Employs demographic forms of similarity (age, Hispanic/Latinx, sex, socioeconomic status, employment status, education level, marital status, strong family structure including multiple generations). Employs psychographic forms of similarity (hobbies, interests, values, lifestyle, symbols, religion, clothing, housing, food, holidays, celebrations, and traditions). Uses clear, simple, familiar words that the audience uses. The language is the primary language of the target audience and this can be English, Spanish, or a combination of both. This should be included in the background graphics and background music. Cultural expressions used including refranes (popular saying traditionally used to express a moral thought, advice, or teaching)	The actor(s), photo(s), voice(s), or video(s) used are dissimilar to the target audience in demographic forms. The actor(s), photo(s), voice(s), or video(s) used are dissimilar to the target audience in psychographic forms. Language is technical, scientific, and complex. The language used is not the primary language of the target audience. Cultural expressions are not used
Call to action	Invokes change and shows this particular audience that they can make a difference (5 points)	Builds awareness or spreads information but fails to invoke change
Gestalt	Target audience generally satisfied and pleased (5 points)	Target audience generally dissatisfied and displeased
Is this message culturally competent?	Interpretation: (1 point per box except the last two are 5 points each). 0-14: Major revisions recommended. 15-19: Minor revisions recommended. 20-25: Culturally competent message	=___/25
*Messages ideally should always be tested with the priority population through qualitative methods like focus groups or listening sessions. All five key areas can be assessed in these conversations, while a call to action and gestalt are critical to obtain from your population.		

In content and clarity, we added attention to the proper use of Spanish accents. Regarding credible sources, we added examples of religious or community leaders, sports athletes, or celebrities. We also added an example regarding color schemes such as colors of the flag or bright colors.

In emotions and values, we gave examples of commonly shared beliefs, including “espiritismo” (the belief that good and evil affect health) or superstition. Other cultural values that could be used for messaging are religion, friendship, “familismo,” and “jeraquismo” (family and hierarchy).

In audience and inclusivity, we emphasized the language used for the message must be the primary language of the target audience, which can be English, Spanish, or a combination of both. This should be included in the background graphics and background music. Additionally, cultural expressions should be used, including “refranes” (popular sayings traditionally used to express a moral thought, advice, or teaching).

In the call to action, the message should invoke change and show the target audience that they can make a difference. In focus groups, interviews, and reviews, we felt there were still intangible characteristics that led some public health messaging to sit well with the target audience while others did not. We allowed for this qualitative interpretation in a category we labeled “gestalt.” In gestalt, the target audience is generally satisfied and pleased.

HOH Guide

For the cultural competency guide for the HOH and deaf community, there also were aspects unchanged from the general and Hispanic guides, including a call to action and gestalt. The aspects unique to public health messaging for this community are detailed below (Table 2, Appendix 3).

**Table 2:** Rubric and guide on cultural competence in public health messaging (deaf/HOH community)

Main rubric components	Do (1 point per box except the last two are 5 points each)	Don't
Content and clarity	Strong and concise message. Demonstrates linguistic fluency. Presents accurate facts with credible sources. Solid background colors with minimal graphic(s) used during the message. Signs at an average of 110 words per minute	Unclear and wordy message. Lack of linguistic fluency. Questionable information or sources. Uses distracting background or image(s) during message. Signing is not between 110-130 words per minute
Emotions and values	Builds on the emotion of trust by clearly displaying the presenter’s titles and credentials (introductions are crucial). The interpreter is of high integrity, producing meaningful and accurate interpretations, and meets advanced interpreter credentials. The interpreter manipulates facial expressions in different degrees to indicate emotional intensity Interpreter is dressed in a solid color shirt that contrasts with skin tone with minimal accessories. Message hones in on the cultural value of collectivism: the close-knit and interconnected group of deaf/HOH	The presenter’s titles and credentials are not visible and the presenter is not sufficiently introduced. The interpreter does not have advanced interpreter credentials leading to subtle or overt inaccuracies in communication. The interpreter lacks physical/facial expression to convey emotion. The interpreter is dressed in patterns or non-contrast patterns with large or distracting accessories. Focuses more on individualism rather than collectivism
Audience and inclusivity	Diversity of the community is shown after message delivery (through graphic(s) or video). Language used takes into consideration the average American adult literacy level. Any background music or slides should be minimal, captions, be placed at a time that is not distracting, and used minimally. ASL, voice-over, and open captions are used. ASL, voice-over, and captions are in sync	Once a message has been delivered, no diversity is shown in following graphic(s) or video). Language is complex, taking into consideration the average American adult has a seventh-/eighth-grade literacy level. No ASL, voice-over, or open captions are used. Signing, voice-over, and captions not in sync
Call to action	Invokes change and shows this particular audience that they can make a difference (5 points)	Builds awareness or spreads information but fails to invoke change
Gestalt	Target audience generally satisfied and pleased (5 points)	Target audience generally dissatisfied and displeased
Is this message culturally competent?	Interpretation: (1 point per box except the last two are 5 points each). 0-14: Major revisions recommended. 15-19: Minor revisions recommended. 20-25: Culturally competent message	=___/25
*Messages ideally should always be tested with the priority population through qualitative methods like focus groups or listening sessions. All 5 key areas can be assessed in these conversations while a call to action and gestalt are critical to obtain from your population.		

In content and clarity, we focus on if the message is delivered with linguistic fluency, defined as ease, confidence, and accuracy with which the target language is used, often ASL. The interpreter must sign at an average of 110 words per minute, and there should be no distracting background images during the message. Despite reviewing national public health messaging designed for this audience, these requirements which may seem simple were often missing and the target audience immediately lost faith in the message.

In emotions and values, this community emphasized that introductions are critical, and, therefore, messaging must build on the emotion of trust by clearly displaying the presenter’s titles and credentials. The interpreter must be of high integrity producing meaningful and accurate interpretation while also manipulating facial expressions in different degrees to indicate emotional intensity. The message must hone in on the cultural value of collectivism for a population that is a very close-knit and interconnected group. The interpreter should not be dressed in non-contrast patterns or large accessories that may be distracting during the signing process.

In audience and inclusivity, we encourage taking into consideration the diversity of the deaf and HOH community in the message. Any background music or slides should be minimal, captioned, and placed at a time that is not distracting. ASL, voice-over, and captions should be used and in sync.

## Discussion

Our findings can help guide more culturally competent public health messaging for minority populations at greater risk of health disparities, such as the Hispanic and HOH communities of South Texas. Our interdisciplinary approach in an underserved region highlights a strategy to address COVID-19 inequities. Our findings add to the body of evidence emphasizing the importance of providing culturally sensitive public health messages, especially to those low-income, uninsured, and indigent, all of which are at a disproportionate risk for COVID-19. The fact that our region of South Texas represented 17% of the state’s COVID-19 deaths while only representing 5% of the state’s population is truly indicative of the healthcare disparities facing Hispanic communities [15]. This led to our medical students, resident physicians and master of social work students, and faculty feeling an urgency to address these disparities, one approach being through culturally competent public health messaging.

Public health messaging has been abundant during the COVID-19 pandemic. The importance of being able to evaluate the effectiveness of delivering the intended messages, as well as monitoring the effect they have on their target audience, is critical in battling healthcare institutional mistrust in minority populations [16]. Public health messages that are created and directed toward White non-Hispanic Americans and native English speakers are not as effective in conveying their message toward minority individuals. Thus, considering a region’s culture and language cannot be stressed enough when creating culturally competent public health messages.

Cultural competence is a complex and dynamic process that identifies and addresses the differences that exist within a group of people [17]. It involves a system that provides care in accordance with the diverse values, beliefs, and ideals of a certain population and then subsequently tailors its effect according to the social, cultural, and linguistic needs of the community [17]. The similarities and differences between how public health messages elicit an emotional response from different groups are key in delivering trusted messages to the communities. Our general cultural competency guide can be further developed for specific target audiences, just as we did with our local Hispanic and HOH communities.

The local safety-net clinic groups played a key role in enabling this project by experiencing the effects of healthcare inequities in the Hispanic population during the COVID-19 crisis. These clinics were the ideal place to develop cultural humility as they provide primary care, integrated mental health care, and various wellness resources to an insured population facing health illiteracy and poverty. We held our focus group meetings with patients served by these clinics to better understand the ground realities, as well as the values held close to the community. Our interdisciplinary team was shocked by how infective the PSAs were in these focus groups, despite preselecting PSAs targeted toward Hispanic communities. Both nationally and regionally created PSAs lacked many of the elements we now describe in our guide to cultural competence. A few of our important learning points included the importance of family and hierarchy, the inclusion of storytelling, and relaying of a positive message in public health messages in order for it to be effective for the Hispanic community. Our qualitative interviews with the HOH community leaders were equally as shocking, with nationally produced PSAs targeting the HOH community not meeting what our team described as key components to establishing rapport with the HOH community. Additionally, the interviews emphasized the values of collectivism and representation. Due to the geographical location, many of our HOH community are also Hispanic, making it no surprise that the top cultural values for each group were “familismo” and “collectivism.” These two terms share very similar meanings, both incorporating the essence of “togetherness.”

While disparities in healthcare have long existed, the COVID-19 pandemic has brought them to the forefront of public health issues [18]. Data from the CDC has further highlighted the disparities in COVID-19 mortality among minorities [19]. Retrospective studies have investigated patients’ comorbidities as well as their social context including resources of neighborhoods’ education, finance, housing, and employment in an attempt to elucidate underlying factors associated with COVID-19 inequities [20]. They found that the incidence of COVID-19 infection was higher in patients with underlying clinical conditions such as diabetes, hypertension, and pulmonary disease [21]. This was of special importance to this project, as the RGV population has the highest rates of diabetes and hypertension in the country, which predisposes this minority population to a higher incidence of clinical disease [21].

The NIH is working to address these issues through the nationwide Community Engagement Alliance Against COVID-19 in Disproportionately Affected Communities (CEAL), which seeks to improve community engagement in COVID-19 prevention, treatment, and vaccine trials [22]. This project, through collaboration with CEAL and UT Health School of Public Health teams, aimed to expand the dissemination of Spanish-language, English, and ASL information beyond the scope of what the CEAL team alone can accomplish through PSAs. These PSAs will educate the RGV’s underserved population about the importance of US minority population participation in COVID-19 prevention, treatment, and vaccine clinical trials and direct them to opportunities to participate for their families’ and communities’ sake, as well as to validate and fully share in the benefits [23]. This transparent, culturally, and contextually grounded approach will enable us to validate community concerns and partner with the community to address our current COVID-19 pandemic and to move the needle in the RGV on health equity and social justice.

This project had its limitations. To comply with the restrictions of the COVID-19 pandemic, we had a small number of ten participants during the in-person focus groups. This restricted the team to reach out en masse to study the target population better. Future projects could include implementing the cultural competency guide with more focus groups and younger/adolescent populations. Another limitation is that we were not able to have a focus group with HOH community members. Although we were able to work extensively with leaders of the HOH community for the creation of the Deaf/HOH public health messaging rubric, we would have liked to meet with individual community members to have them assess and evaluate PSAs similar to what we did for the Hispanic community.

## Conclusions

The devastating effects of the COVID-19 pandemic have shed light on the existing inequities in the community but have also provided an opportunity to address these disparities. Effective and culturally competent public health messages can help combat testing fears, improve contact tracing, motivate individuals to participate in trials, and vaccinations, and ultimately aid to address the rampant COVID-19 inequities that exist in minority communities. Thus, we hope that these guides our interdisciplinary team developed can be used to create PSAs that are culturally competent, either by use of our general guide, Hispanic guide, or HOH guide. Our lessons learned in public health messaging with the deaf and HOH community would be applicable to the 1 million deaf people who use ASL as their primary language. Additionally, addressing the needs of the Hispanic population will affect the 41 million native Spanish speakers, and having culturally competent public health messaging is critical and of national concern.

## Figures and Tables

**Figure 2:**
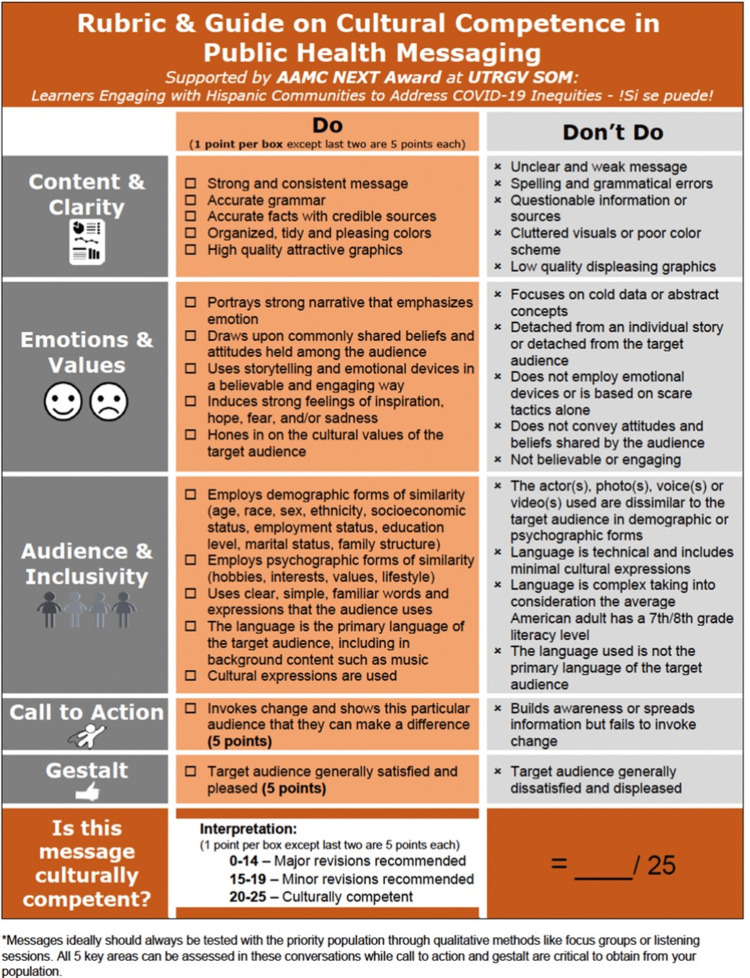
Rubric and guide on cultural competence in public health messaging

**Figure 3:**
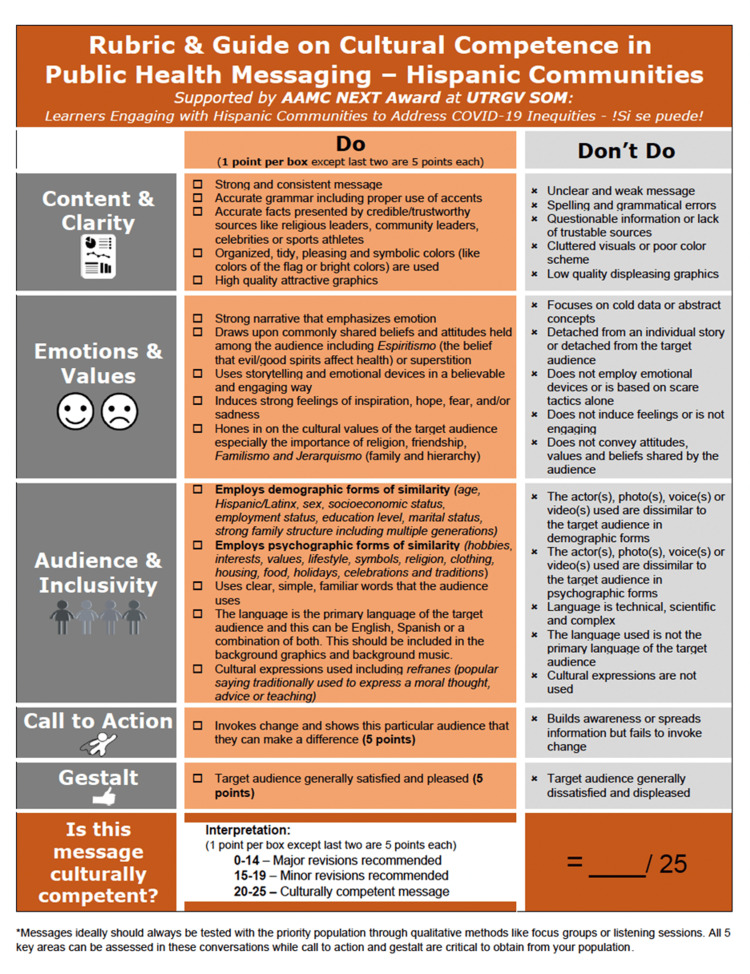
Rubric and guide on cultural competence in public health messaging (Hispanic communities)

**Figure 4:**
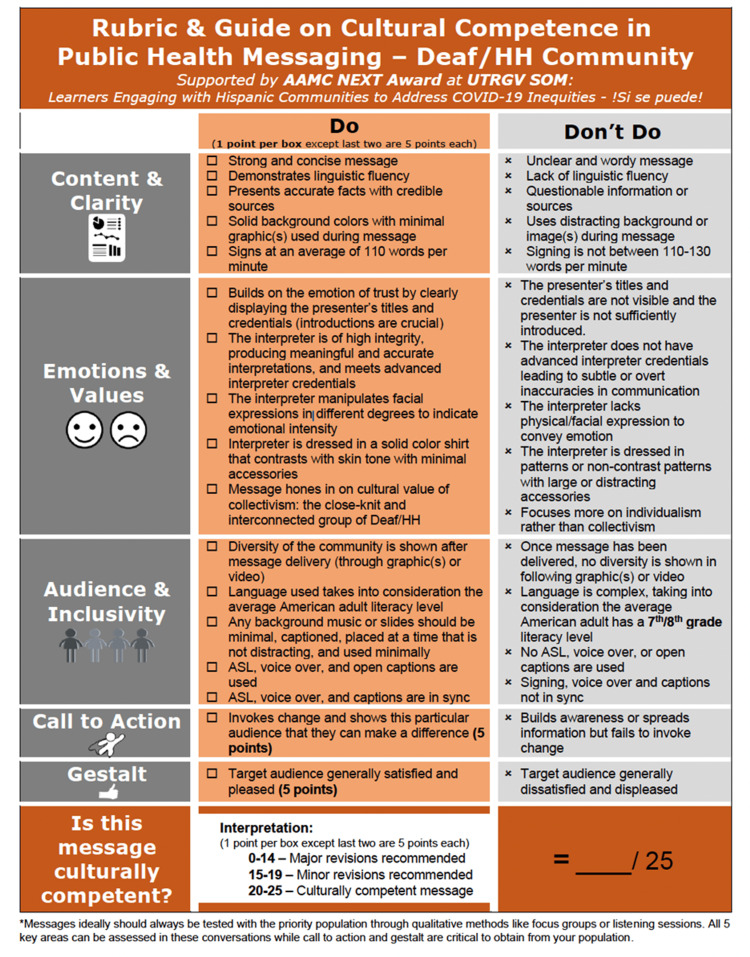
Rubric and guide on cultural competence in public health messaging (deaf/HOH community)
